# Physical fitness and white matter microstructure in children with overweight or obesity: the ActiveBrains project

**DOI:** 10.1038/s41598-020-67996-2

**Published:** 2020-07-27

**Authors:** M. Rodriguez-Ayllon, I. Esteban-Cornejo, J. Verdejo-Román, R. L. Muetzel, J. Mora-Gonzalez, C. Cadenas-Sanchez, A. Plaza-Florido, P. Molina-Garcia, A. F. Kramer, A. Catena, F. B. Ortega

**Affiliations:** 1 PROFITH “Promoting Fitness and Health Through Physical Activity” Research Group, Department of Physical and Sports Education, Faculty of Sport Sciences, University of Granada, Carretera de Alfacar, s/n, 18071 Granada, Spain; 2 The Brain, Mind and Behavior Research Center, University of Granada (CIMCYC-UGR), Granada, Spain; 3 Laboratory of Cognitive and Computational Neuroscience (UCM-UPM), Centre for Biomedical Technology (CTB), Madrid, Spain; 4 Department of Child and Adolescent Psychiatry, Erasmus MC – Sophia Children’s Hospital, Rotterdam, The Netherlands; 5 Department of Epidemiology, Erasmus MC, Rotterdam, The Netherlands; 6 College of Health and Human Services, University of North Carolina at Charlotte, Charlotte, NC USA; 7 MOVE-IT Research Group and Department of Physical Education, Faculty of Education Sciences, University of Cádiz, Cádiz, Spain; 8 Biomedical Research and Innovation Institute of Cádiz (INiBICA) Research Unit, Puerta del Mar University Hospital, University of Cádiz, Cádiz, Spain; 9 Center for Cognitive and Brain Health, Department of Psychology, Northeastern University, Boston, MA USA; 10 Beckman Institute, University of Illinois at Urbana-Champaign, Urbana, IL USA; 11 Department of Clinical Psychology, University of Granada, Granada, Spain; 12 Department of Biosciences and Nutrition, Karolinska Institutet, Group MLO, 14183 Huddinge, Sweden

**Keywords:** Paediatric research, Risk factors, Psychology and behaviour, Neuronal development

## Abstract

Recent studies investigated the association of cardiorespiratory fitness with white matter microstructure in children, yet little work has explored to what extent other components of physical fitness (i.e., muscular or motor fitness) are associated with white matter microstructure. Indeed, this association has not been previously explored in children with overweight/obesity who present a different white matter development. Therefore, we aimed to examine associations between physical fitness components and white matter microstructure in children with overweight/obesity. In total, 104 (10.04 ± 1.15 years old; 43 girls) children were included in this cross-sectional study. Physical fitness was assessed using the ALPHA-fitness test battery. Fractional anisotropy (FA) and mean diffusivity were derived from diffusion tensor imaging (DTI). No association was found between physical fitness and global DTI metrics (all *P* > 0.082). Within individual tracts, all associations became non-significant when analyses were adjusted for multiple comparisons. Using the voxel-wise approach, we identified a small cluster in the left lateral frontal lobe where children with greater upper-body muscular fitness showed higher FA (P_*FWE-corrected*_ = 0.042). Although our results cannot conclude physical fitness is related to white matter microstructure in children with overweight/obesity; those findings indicate that the association of muscular fitness with white matter microstructure might be more focal on frontal areas of the brain, as opposed to global differences.

## Introduction

Childhood is a critical period for neurodevelopment^1^, especially sensitive to a number of health-related factors that could have an influence on the brain^2,3^. In particular, physical fitness (i.e., the capacity to perform physical activity) is considered a powerful marker of health in children and adolescents^4^. Physical fitness is composed of a set of physical components such as cardiorespiratory fitness (i.e. the capacity of the cardiovascular and respiratory systems to carry out prolonged strenuous exercise), muscular fitness (i.e. the capacity to exert work against a resistance), and motor fitness (i.e. the ability to move the body as fast as possible)^4^. Previous evidence suggests that higher physical fitness levels are positively associated with a better physical and mental health in children and adolescents, both immediately^4, 5^ and later in life^6,7^. In addition, recent research has also shed light on the positive role of physical fitness on brain health. For instance, an American College of Sports Medicine (ACSM) Position Stand based on physical fitness and brain suggested that physical fitness may have a positive influence on brain structure in children^2^. However, few studies have explored the extent to which physical fitness is associated with white matter microstructure during childhood^8,9^.

White matter development includes further axon myelination via thickening of the myelin sheaths, axonal growth, and increasing calibre of fibre tracts^10^. Particularly, white matter is required for efficient transmission of information between brain areas into structural networks to support cognitive function and mental health^11,12^. To date, only two studies have examined the association between physical fitness and white matter microstructure in young people^8,9^. Specifically, cardiorespiratory fitness was positively associated with white matter microstructure (i.e., corpus callosum, corona radiata and superior longitudinal fasciculus (SLF)) in children^8^; whereas among, adolescents, cardiorespiratory fitness was negatively associated with white matter microstructure in the corticospinal tract (CST)^9^. Apart from cardiorespiratory fitness, there are two other physical fitness components (i.e., muscular fitness and motor fitness) that have been proven to differentially influence physical and brain health during childhood^4,13^. However, previous studies addressing the relationship between physical fitness and white matter microstructure only focused on cardiorespiratory fitness and did not examine muscular fitness or motor fitness.

Lastly, previous literature suggests that excess body mass has been linked to a different structural connectivity^14^ and white matter^15^ development in children. For instance, compared with normal weight children, obese children showed differences in white matter organization, mainly in frontal and temporal brain regions^15^. However, association between physical fitness and white matter microstructure, in pediatric populations, has only been studied in normal-weight youths^8,9^. Taking into account the lack of studies testing the association between different components of physical fitness and white matter microstructure in children, as well as, the previously observed white matter differences in children with overweight or obesity, there is a clear need for studies that examine how different components of physical fitness (i.e., cardiorespiratory fitness, muscular fitness and motor fitness) associate with white matter microstructure in younger populations, and particularly in children with overweight or obesity.

Therefore, the aim of the present study was to examine the associations of components of physical fitness with white matter microstructure in children with overweight or obesity. On the basis of previous literature^8,9^, the general hypothesis was that higher levels of physical fitness would relate to greater white matter microstructure in children with overweight or obesity. However, a specific hypothesis about the potential strength or anatomical location of these relationships remained open.

## Materials and methods

### Study design and participants

This cross-sectional study is part of the ActiveBrains project (https://profith.ugr.es/activebrains?lang=en), a randomized controlled trial, with the primary aim of examining the effects of exercise on brain, cognition and academic performance in children with overweight or obesity according to sex and age specific World Obesity Federation cut-off points^16,17^. The complete methodology of the project has been described elsewhere^18^. In total, 110 children with overweight or obesity, ages 8-to-11 years, were recruited from Granada (southern Spain). Of these, 104 (10.04 ± 1.15 years old; 43 girls) were included in the present analyses. Data were collected from November 2014 to February 2016. Parents or legal guardians were informed of the goal of the study and written informed parental and child consents were obtained. This study was conducted according to the Declaration of Helsinki, approved by the Human Research Ethics Committee of the University of Granada, and registered in ClinicalTrials.gov (identifier: NCT02295072).

### Physical fitness components and magnetic resonance imaging (MRI) procedure

#### Physical fitness components

Physical fitness components (i.e., cardiorespiratory fitness, muscular fitness, and motor fitness) were assessed using the extended version of the ALPHA (Assessing Levels of Physical fitness and Health in Adolescents) health-related physical fitness test battery^19^. This battery has been shown to be valid, reliable, feasible, and safe for the assessment of the physical fitness components in children and adolescents^19^.

Cardiorespiratory fitness was estimated by the 20-m shuttle-run test^20^. This test was always performed at the end of the fitness battery testing session. The total number of completed laps were registered. Upper- and lower-body muscular fitness were assessed using the handgrip strength test and the standing long jump test, respectively. A digital hand dynamometer with an adjustable grip (TKK 5101 Grip D, Takei, Tokyo, Japan) was used to assess upper-body muscular fitness. Each child performed the test twice, and the maximum scores of left and right hands were averaged and used as a measurement of absolute upper-body muscular fitness in kilograms (kg). The standing long jump test was performed three times and the longest jump was recorded in centimeters (cm) as a measurement of relative lower-body muscular fitness. In addition, we computed a relative-to-body weight measurement from upper body muscular fitness (kg/body weight) and an absolute measurement from lower body muscular fitness (cm * kg), according to previous research in children with obesity^21^. Motor fitness was assessed using the 4 × 10-m shuttle-run test. Participants were required to run back and forth twice between two lines 10-m apart. Children were instructed to run as fast as possible and every time they crossed any of the lines, they were instructed to pick up (the first time) or exchange (second and third time) a sponge that had earlier been placed behind the lines. The test was performed twice and the fastest time was recorded in seconds. Since a longer completion time indicates a lower fitness level, for analysis purposes we inverted this variable by multiplying test completion time (s) by − 1. Thus, higher scores indicated higher motor fitness levels.

#### Image acquisition

MRI data were collected with a 3.0 T Siemens Magnetom Tim Trio scanner (Siemens Medical Solutions, Erlangen, Germany). Diffusion tensor imaging (DTI) data were acquired using an echo planar imaging (EPI) sequence with the following parameters: repetition time (TR) = 3,300 ms, echo time (TE) = 90 ms, flip angle = 90, matrix = 128 × 128, field of view (FOV) = 230 mm × 230 mm, slice thickness = 4 mm, number of slices = 25 and voxel resolution = 1.8 × 1.8 × 4 mm^3^. One volume without diffusion weighting (b = 0 s/mm^2^) and 30 volumes with diffusion weighting (b = 1000 s/mm^2^) were collected.

#### Image preprocessing

DTI is able to sample features of the microstructural architecture of white matter^22^. To quantify total DTI metrics, we use fractional anisotropy (FA) and mean diffusivity (MD), as two of the most common derived scalar metrics from DTI^23^. FA expresses the degree to which water diffuses preferentially along one axis, and has shown to increase with age^23^ during development and to be lower in the context of various neurological and psychiatric diseases^24^. MD is a scalar describing the average diffusion in all directions, with higher levels indicating relatively unimpeded diffusion (i.e., negatively correlated with FA)^25^.

Functional MRI of the Brain Software Library (FSL) (https://fsl.fmrib.ox.ac.uk) was used to processed MRI data^26,27^. First, images were adjusted for minor head motion^28^, which included a Gaussian process for outlier replacement^29^. Then, the resulting transformation matrices were used to rotate the diffusion gradient direction table^30,31^. Non-brain tissue was removed using the FSL Brain Extraction Tool^32^. Lastly, the diffusion tensor was fit, and common scalar maps (i.e., FA and MD) were subsequently computed.

#### Probabilistic fiber tractography

Fully automated probabilistic fiber tractography was performed using the FSL plugin, “AutoPtx” (https://fsl.fmrib.ox.ac.uk/fsl/fslwiki/AutoPtx). Diffusion data were processed using the Bayesian Estimation of Diffusion Parameters Obtained using Sampling Techniques (BEDPOSTx), accounting for two fiber orientations at each voxel^33,34^. Then, for each subject, the FA map was aligned to the FMRIB-58 FA template image with the FSL nonlinear registration tool (FNIRT). Next, the inverse of this nonlinear warp field was computed, and applied to a series of predefined seed, target, exclusion, and termination masks provided by the AutoPtx plugin^35^. Probabilistic fiber tracking was then execute with the FSL Probtrackx module using these supplied tract-specific masks (i.e., seed, target, etc.) that were warped to the native diffusion image space of each subject^33^. Lastly, the resulting path distributions were normalized to a scale from 0 to 1 using the total number of successful seed-to-target attempts and were subsequently thresholded to remove low-probability voxels likely related to noise.

White matter tract segmentation was performed by thresholding the normalized tract density images based on previously established values by de Groot et al.^35^ (i.e., cingulate gyrus part of cingulum (CGC): 0.01, CST: 0.005, forceps major (FMA): 0.005, forceps minor (FMI): 0.01, inferior longitudinal fasciculus (ILF): 0.005, SLF: 0.001, uncinate fasciculus (UNC): 0.01). Average FA and MD values were then computed for each fiber bundle. Connectivity distributions were estimated for the 7 large fiber bundles previously named and selected based on previous reports^36–38^. Average of FA and MD in the left and right hemisphere was calculated in those tracts present in both hemispheres (i.e., CGC, CST, FMA, FMI, ILF, SLF, and UNC).

To assess whether physical fitness components (i.e., cardiorespiratory fitness, muscular fitness, and motor fitness) were related to global measures of white matter microstructure (i.e., global FA, MD), selected tracts were combined into a single factor (“global factor”). The global factor was computed by averaging all tracts and weighting this average by the size (volume) of the tracts.

#### Tract-based spatial statistics

Tract-based spatial statistics (TBSS) was used to perform voxel-wise statistical analyses of the DTI data (https://fsl.fmrib.ox.ac.uk/fsl/fslwiki/TBSS/UserGuide^39^. A mean FA image was calculated and thinned to create a mean FA skeleton, which represents the center of white matter tracts. A threshold of FA > 0.2 was selected to exclude voxels not belonging to white matter. FA maps of each participant were then projected onto the skeleton. The same procedure was applied to the MD maps.

#### Image quality assurance

Raw image quality was assessed via visual inspection. In addition, the sum-of-squares error (SSE) maps from the tensor estimation were calculated and visually inspected for structured noise^12^. Image quality was rated using a 4-point scale, with 1 = “excellent”, 2 = “minor”, 3 = “moderate”, and 4 = “severe”. Datasets determined to be of insufficient quality (i.e., moderate and severe) for statistical analyses were excluded (n = 2). Lastly, probabilistic tractography data were inspected visually. First, the native space FA map registration was inspected to ensure images were all properly aligned to the template (masks were properly mapped to native space). Second, all tracts were visualized to ensure accurate path reconstruction.

### Covariates

Body weight and height were performed with participants having bare feet and wearing underclothes; weight was measured with an electronic scale (SECA 861, Hamburg, Germany) and height (cm) with a stadiometer (SECA 225, Hamburg, Germany). Both measurements were performed twice, and averages were used. BMI was expressed in kg/m^2^. PHV is a common indicator of maturity in children and adolescents^40^. PHV was obtained from anthropometric variables (weight, height and/or seated height) using Moore’s equations^41^. The total composite IQ was assessed by the Spanish version of the Kaufman Brief Intelligence Test (K-BIT), a validated and reliable instrument^42^. This test consists of vocabulary and matrices subtests which provided indicators of crystallized intelligence and fluid intelligence, respectively. The typical punctuation of both, crystallized and fluid indicators of intelligence, were computed and a total intelligence score was obtained from the sum of them. Parental education was assessed by the educational level of mother and father reported (i.e., no elementary school, elementary school, middle school, high school and university completed). Parent answers were combined into a trichotomous variable (i.e., none of the parents had a university degree, one of the parents had a university degree and both parents had a university degree). Lastly, the Behavior Assessment System for Children (BASC), level-2 for children aged 6–12 years old, was used to assess behavioral and emotional functioning. A total behavioral symptoms index (including aggressively, hyperactivity, attention problems, atypical behaviors, anxiety and depression) was extracted from the questionnaire^43^.

### Statistical analysis

All analyses, with the exception of TBSS analyses, were performed using the Statistical Package for Social Sciences (IBM SPSS Statistics for Windows, version 22.0, Armonk, NY, *P* set at < 0.05). The characteristics of the study sample are presented as means and standard deviations (SD) or percentages. In addition, we tested the correlation of BMI with global DTI metrics and physical fitness components. Interaction analyses of sex with physical fitness variables were also performed. No significant interactions with sex were found (*P* ≥ 0.10) and therefore analyses are presented for the whole sample. In addition, we explored the association of several confounders (i.e., sex, PHV, BMI, IQ, parental education, and emotional and behavioral problems) with tractography-derived white matter variables using a Pearson's bivariate correlation analysis (data no shown). Among all of the potential confounders, parental education, socioeconomic status, and emotional and behavioral problems were not significantly related to white matter microstructure (all *P* values > 0.1) and were therefore excluded from the subsequent analyses.

Separate linear regression analyses adjusted for sex, PHV, BMI and IQ were performed to examine the association between physical fitness components and global-extracted DTI scalar metrics (i.e., global FA and MD). Each regression model examined separately the relationships between a single physical fitness component and a single DTI scalar metric.

Then, in order to determine whether the association of physical fitness with white matter microstructure was indeed only global or restricted to a particular set of white matter bundles, and to facilitate comparison with future studies, we applied two commonly used methodologies: (1) probabilistic tractography of large, commonly studied white matter tracts and (2) TBSS, which is a voxel-based approach. For probabilistic tractography analyses, false discovery rate (FDR. Benjamini–Hochberg method) was used to adjust for multiple comparisons^44^. Correction for multiple comparisons was based on 7 tracts, 2 DTI metrics and 6 physical fitness components for a total of 84 tests. For TBSS analyses, the association between physical fitness components and DTI scalar metrics were tested voxel-wise using general linear models, including sex, PHV, BMI and IQ as covariates. A permutation-based statistical approach (5,000 permutations) within FSL's Randomise^39^ was performed including the threshold-free cluster enhancement (TFCE) multiple comparison correction method. Significance was set at *P* < 0.05, corrected for family-wise error.

## Results

Table 1 presents demographic participant characteristics. No correlation was found between BMI and global DTI metrics. However, a higher BMI was correlated with a lower cardiorespiratory fitness, motor fitness and relative muscular fitness (r ranges from − 0.490 to − 0.341). Of note, BMI was positively correlated with absolute muscular fitness, including upper-body muscular fitness (r = 0.276) and lower-body muscular fitness (r = 0.372). The association between physical fitness components (i.e., cardiorespiratory fitness, muscular fitness, and motor fitness) and global FA and global MD is shown in Table 2. Briefly, no associations were found between physical fitness components and any of the global white matter metrics (i.e., FA and MD) (all *P* values > 0.05).

**Table 1:** Descriptive sample characteristics.

	Mean/%	SD
**Sex**		
Girls,%	41.35	
Age (years)	10.04	± 1.15
Peak height velocity (years)	− 1.90	± 1.04
Body Mass Index	26.68	± 3.63
**Body Mass Index, %**		
Overweight	26.9	
Obesity type I	43.3	
Obesity type II	29.8	
**Parental education university level (%)**		
Neither parent	63.46	
One parent	19.23	
Both parents	17.31	
Intelligence (Test-KBIT)	48.10	± 24.97
**Physical fitness components**		
Cardiorespiratory fitness		
Last completed lap (20 m shuttle run)	16.14	± 7.88
Muscular fitness		
Relative upper-body muscular fitness (kg/kg)	0.30	± 0.06
Relative lower-body muscular fitness (cm)	104.95	± 18.64
Absolute upper-body muscular fitness (kg)	16.77	± 4.22
Absolute lower-body muscular fitness (cm × kg)	5,825.13	± 1,450.83
Motor fitness (s)	15.12	± 1.60

Values are expressed as means ± standard deviations, unless otherwise indicated. Test-KBIT = The Kaufman Brief Intelligence Test.

**Table 2:** Association of physical fitness components with global FA, and global MD in children with overweight/obesity (n = 89).

	Global FA		Global MD	
	β	*P*	β	*P*
Cardiorespiratory fitness (laps)	0.104	0.473	− 0.026	0.851
Relative upper-body muscular fitness (kg/kg)	0.131	0.328	− 0.155	0.225
Relative lower-body muscular fitness (cm)	− 0.173	0.178	0.209	0.085
Absolute upper-body muscular fitness (kg)	0.114	0.452	− 0.221	0.127
Absolute lower-body muscular fitness (cm × kg)	− 0.279	0.099	0.278	0.082
Motor fitness (s^−1^)	− 0.089	0.547	0.022	0.876

Lineal regression model was adjusted for sex, peak height velocity, body mass index (kg/m^2^) and intelligence quotient. FA = Fractional anisotropy (high FA corresponds to preferential diffusion along one direction an indication a high level of tissue organization), MD = mean diffusivity (high MD corresponds to relatively unimpeded water diffusion and indicates regions of low tissue organization). Values are standardized regression coefficients (β).

Association between physical fitness components and tract-specific FA and MD is shown in Table 3. Cardiorespiratory fitness was positively associated with FA in the ILF (β = 0.273, *P* = 0.039). In addition, relative upper-body muscular fitness was negatively associated with MD in the ILF (β = − 0.237, *P* = 0.035). All these associations became non-significant when analyses were adjusted for multiple comparisons (all *P* values > 0.05). No association was found between motor fitness and tract-specific FA and MD (all *P* values > 0.05).

**Table 3:** Association of physical fitness components and tract-specific FA and MD in children with overweight or obesity.

	Cardiorespiratory fitness		Relative upper-body MF		Relative lower-body MF		Absolute upper-body MF		Absolute lower-body MF		Motor fitness	
	FA	MD	FA	MD	FA	MD	FA	MD	FA	MD	FA	MD
CGC ^a^	− 0.126	− 0.107	− 0.010	− 0.155	− 0.116	0.070	− 0.064	− 0.154	− 0.159	0.181	− 0.234	0.003
CST	0.100	− 0.089	− 0.011	− 0.106	− 0.098	0.047	− 0.003	− 0.108	− 0.106	0.084	0.032	− 0.072
ILF	0.273*	− 0.103	***0.212***	− 0.237*	0.024	0.084	0.159	*** − 0.214***	− 0.075	0.205	0.151	− 0.040
SLF	0.119	0.006	0.152	− 0.138	− 0.048	0.111	0.197	*** − 0.236***	− 0.056	0.080	0.056	− 0.026
UNC	− 0.010	− 0.060	0.006	− 0.081	0.050	0.029	− 0.052	− 0.089	0.050	0.062	− 0.021	− 0.016
FMA	0.182	− 0.023	0.106	0.062	− 0.122	0.183	0.057	0.032	*** − 0.270***	***0.272***	0.090	0.004
FMI	0.129	− 0.151	− 0.120	0.114	− 0.075	0.100	− 0.152	0.069	− 0.164	0.086	− 0.079	− 0.070

Lineal regression model was adjusted for sex, peak height velocity, body mass index (kg/m^2^) and intelligence quotient. FA = Fractional anisotropy (high FA corresponds to preferential diffusion along one direction an indication a high level of tissue organization), MD = mean diffusivity (high MD corresponds to relatively unimpeded water diffusion and indicates regions of low tissue organization). MF = Muscular fitness. Cingulate gyrus part of cingulum (CGC), corticospinal tract (CST), inferior longitudinal fasciculus (ILF), superior longitudinal fasciculus (SLF), uncinate fasciculus (UNC), forceps major (FMA), and forceps minor (FMI). Values are standardized regression coefficients (β). Statistically significant values are shown in bold (*P* < 0.05), and borderline significant values are shown in italics and bold (*P* < 0.1). *P < 0.05. All the associations showed in the table disappeared when analyses were adjusted for multiple comparisons. ^a^In the cingulate gyrus part of cingulum n = 89.

**Figure 1:**
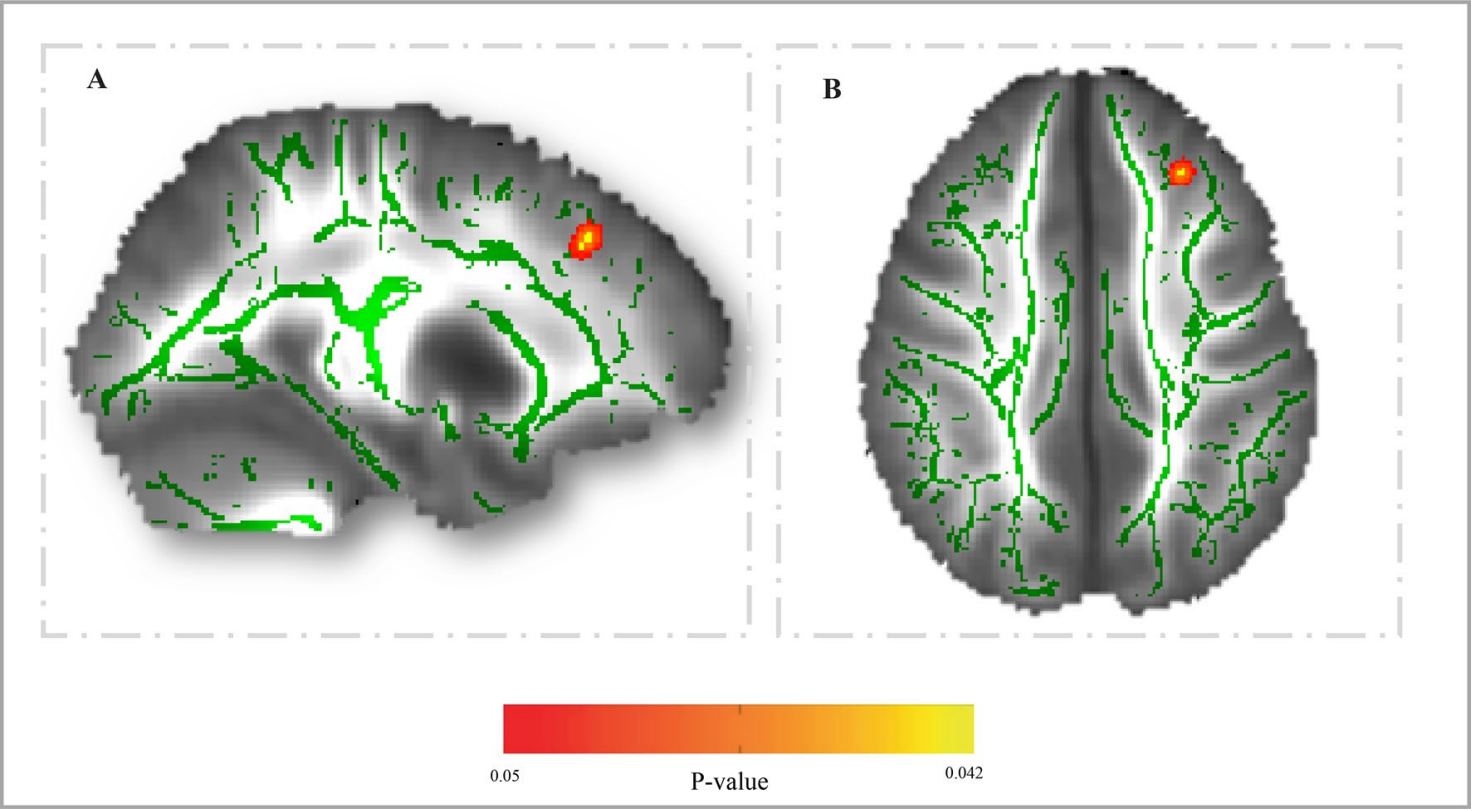
Positive association between absolute upper-body muscular fitness and FA in the left lateral frontal lobe (Montreal Neurological Institute—MNI-coordinates x = − 25, y = 30, z = 34; cluster size = 13; corrected *P* = 0.042). A = sagittal view and B = axial view. The colour bar represents *P* values, with yellow colour indicating higher significant association. The left hemisphere corresponds to the right side of the axial view.

Figure 1 presents the results of the voxel-wise DTI parameter analyses (i.e. TBSS). A statistically significant positive association between absolute upper-body muscular fitness and FA in the left lateral frontal lobe (X_MNI_ = − 25, Y = 30, Z = 34, cluster size = 13, P_*FWE-corrected*_ = 0.042) was found after correction for multiple comparisons.

## Discussion

The aim of the present study was to examine the associations of physical fitness components (i.e., cardiorespiratory fitness, muscular fitness, and motor fitness) with white matter microstructure in children with overweight or obesity. No significant association was found between physical fitness components and global DTI scalar metrics (i.e., global FA, and global MD). Within individual tracts, all associations became non-significant when analyses were adjusted for multiple comparisons. However, results of the voxel-wise DTI parameter analyses showed that absolute upper-body muscular fitness was positively associated with FA in the left lateral frontal lobe after adjusting for multiple comparisons.

Cardiorespiratory fitness was not related to global metrics of DTI (i.e., global FA, and global MD) in children with overweight or obesity. The current study found that cardiorespiratory fitness was positively associated with FA in the ILF, although this association became non-significant when analyses were adjusted for multiple comparisons. These findings were consistent with the TBBS analyses showing no association with cardiorespiratory fitness. Chaddock-Heyman et al.^8^, using data from the FITKids project, found that higher levels of cardiorespiratory fitness were associated with greater FA in sections of the corpus callosum, corona radiata and SLF in children. These associated tracts differ from our results and may be partially explained by the different analysis approach used (i.e., Chaddock et al. used region-of-interest analyses vs. our study that used data-driven analysis adjusted for multiple comparison). In addition, measurement differences differ between the studies (e.g., different methodology of cardiorespiratory fitness assessment, differences among the MRI scanners or the MRI sequence acquisition parameters, participant demographics, participants BMI, etc.). Of note, our group, using data from the ActiveBrains and FITKids project, recently published that the white matter brain regions volumes associated with cardiorespiratory fitness were mainly located in the SLF and the ILF in children with overweight or obesity of the same approximate ages^45^. Consequently, although our results cannot conclude that cardiorespiratory fitness is related to white matter microstructure in children with overweight or obesity, these results, in line with previous literature^8^, seem to indicate that the association between cardiorespiratory fitness and white matter microstructure in children with overweight or obesity might be more focal than global white matter, and related to long association fiber tracts.

Regarding the other two physical fitness components, both muscular and motor fitness were not related to global metrics of DTI (i.e., global FA, and global MD) in children with overweight or obesity. In addition, while motor fitness was not associated with tract-specific white matter microstructure, relative upper-body muscular fitness was negatively associated with MD in the ILF. Of note, this association became non-significant when analyses were adjusted for multiple comparisons. However, when using the TBSS approach, we identified a small cluster in the left lateral frontal lobe where children with greater absolute upper-body muscular fitness showed higher FA, after adjusting for multiple comparison. Previous literature regarding both muscular and motor fitness in relation to white matter microstructure is not available, which hampers direct comparisons with other studies. Nonetheless, in line with our results, previous studies found that adolescents with higher muscular fitness, specifically upper-body muscular fitness, had a 20–30% lower risk of death from suicide and were 15–65% less likely to have any psychiatric diagnosis such as schizophrenia and mood disorders^46^. In addition, it was found that higher muscular fitness during adolescence predicts lower risk of obtaining disability pension due to all causes^7^. Of note, our group recently published that higher upper-body muscular fitness was negatively associated with stress and negative affect, and positively associated with self-esteem in children with overweight or obesity^47^. Therefore, we speculate that muscular fitness plays an undefined role in white matter microstructure which in turn could mediate or moderate mental health. Future work with a larger sample should confirm or contrast this hypothesis.

The underlying plausible mechanisms of the role of muscular fitness on white matter in children with overweight or obesity cannot be elucidated in our study. However, previous literature suggested that muscle contraction induced peripheral factors (e.g., irisin, and cathepsin B) which passes through the blood–brain barrier to enhance brain-derived neurotrophic factors and hence neurogenesis, memory and learning^48^. However, it is unknown whether this myokine is a determining factor in muscle-induced enhanced white matter microstructure in children. It has been also suggested that exercised skeletal muscle leads to upregulation of PGC1α in mouse model and human skeletal muscle cells^49^. Likewise, endurance exercise training can lead to activation of the PGC1α, which stimulates the expression of kynurenine aminotransferase within skeletal muscle^49^. Moreover, higher expression of kynurenine aminotransferase can lead to increased conversion of neurotoxic kynurenine into neuroprotective kynurenic acid. The fact that kynurenic acid is not able to cross the blood–brain barrier protects the brain from stress-induced kynurenine accumulation, neuroinflammation and changes in synaptic plasticity. Therefore, although much still needs to be explored about the mechanisms that explain a relationship between muscular fitness and white matter microstructure in children, based on previous evidence in animal models, the positive associations between upper-body muscular fitness and greater FA in the frontal lobe is neurologically and biologically plausible.

The limitations of this study include (1) its cross-sectional design, which does not allow us to draw causal associations; (2) our focus on children with overweight or obesity, which limits the generalizability of our findings to the entire range of the BMI distribution; (3) the relatively small sample size, which could explain the few associations found in the analyses, although the sample size is respectable for neuroimaging studies in children; (4) and the voxel size which was a 4-mm-section nonisotropic voxel (1.8 × 1.8 × 4 mm^3^). Therefore, FA could be underestimated in regions containing crossing fibers (i.e., SLF). On the other hand, the FA measured in regions without crossing fibers (i.e., CST) is not prone to underestimation^50^. Lastly, the effect sizes for the association between physical fitness components and white matter microstructure were statistically non-significant or relatively small. Larger effects may not be expected due to the preservation of white matter microstructural development in the majority of young people, which probably has not yet achieved the maturational peak in most of the tracts^23^. Key strengths of the current study are the inclusion of neuroimaging data, and the combination of probabilistic fiber tractography and voxel-wise analyses of white matter tracts.

## Conclusion

We found that physical fitness components are not associated with global DTI metrics (i.e., global FA, and global MD). Within individual tracts, all associations became non-significant when analyses were adjusted for multiple comparisons. However, using the TBSS approach, we identified a small cluster in the left lateral frontal lobe where children with greater absolute upper-body muscular fitness showed higher FA, after adjusting for multiple comparison. Our results cannot conclude that physical fitness components are related to white matter microstructure; however, the results seem to indicate that the association between physical fitness components (i.e., specifically muscular fitness) and white matter microstructure is more focal on specific tracts, as opposed to global differences. Future longitudinal and randomized control trials should explore the role of different physical fitness components on white matter microstructure.
